# Comparing individual and population measures of senescence across 10 years in a wild insect population

**DOI:** 10.1111/evo.13674

**Published:** 2019-01-10

**Authors:** Rolando Rodríguez‐Muñoz, Jelle J. Boonekamp, Xing P. Liu, Ian Skicko, Sophie Haugland Pedersen, David N. Fisher, Paul Hopwood, Tom Tregenza

**Affiliations:** ^1^ School of Biosciences, Centre for Ecology & Conservation University of Exeter Penryn Campus TR10 9FE United Kingdom; ^2^ College of Forestry Jiangxi Agricultural University Nanchang 330045 Jiangxi China; ^3^ Department of Psychology, Neuroscience & Behaviour McMaster University 1280 Main St West Hamilton Ontario L8S 4L8 Canada

**Keywords:** BaSTA, demographic ageing, extrinsic mortality, intrinsic mortality, longevity, longitudinal study

## Abstract

Declines in survival and performance with advancing age (senescence) have been widely documented in natural populations, but whether patterns of senescence across traits reflect a common underlying process of biological ageing remains unclear. Senescence is typically characterized via assessments of the rate of change in mortality with age (actuarial senescence) or the rate of change in phenotypic performance with age (phenotypic senescence). Although both phenomena are considered indicative of underlying declines in somatic integrity, whether actuarial and phenotypic senescence rates are actually correlated has yet to be established. Here we present evidence of both actuarial and phenotypic senescence from a decade‐long longitudinal field study of wild insects. By tagging every individual and using continuous video monitoring with a network of up to 140 video cameras, we were able to record survival and behavioral data on an entire adult population of field crickets. This reveals that both actuarial and phenotypic senescence vary substantially across 10 annual generations. This variation allows us to identify a strong correlation between actuarial and phenotypic measures of senescence. Our study demonstrates age‐related phenotypic declines reflected in population level mortality rates and reveals that observations of senescence in a single year may not be representative of a general pattern.

There is a broad consensus that senescence, “the age‐related decline in fitness traits that arises due to internal physiological deterioration” (Rose 1991), is widespread in natural populations. This conclusion has largely been reached on the basis of measurements of demographic variables, usually longevity and fecundity, although there are a growing number of studies in which phenotypic traits are measured across individual lifespans (Nussey et al. 2013). New methods have been developed to fit parametric mortality functions (Colchero et al. 2012), which have proved to be valuable for deriving demographic measures of senescence without the necessity of collecting longitudinal samples of individuals to track senescence at the individual phenotypic level (e.g., Zajitschek et al. 2009a; Warner et al. 2016). However, longitudinal studies of wild vertebrates have identified substantial heterogeneity in the pattern of phenotypic senescence among traits (Nussey et al. 2009; Hayward et al. 2015), raising the question of the extent to which single phenotypic traits can be expected to be related to demographic patterns such as actuarial senescence. Variation in the intensity of senescence has been understood in the context of an adaptive life‐history in which resources that could be used to maintain body condition in later life are instead used to increase reproductive output earlier in life (Williams 1957; Kirkwood and Holliday 1979; Partridge and Barton 1993). This adaptive life‐history theory of ageing predicts that patterns of senescence should be affected by environmental factors that impinge on trade‐offs between allocation to reproduction early and late in life. Hence, we assume that the ultimate explanation for the observed differences in patterns of senescence lies in differential resource allocation among traits and their respective fitness returns on investment (Lemaître et al. 2015). Traits that are relatively unimportant for fitness might tend to senesce at a faster rate because declines in these traits would incur a smaller fitness penalty. However, the pattern of optimal resource allocation among traits related to survival and reproduction remains difficult to predict due to the paucity of knowledge about the underlying physiological mechanisms. Consequently, we lack a predictive framework linking trajectories of phenotypic and actuarial senescence, highlighting the importance of direct comparisons.

Beyond the functional explanations for asynchrony of senescence, there are also statistical factors that may lead to a mismatch between actuarial and phenotypic measures of senescence, even when they would be similarly influenced by physiological deterioration. A common approach to measuring actuarial senescence is to fit demographic data to parametric mortality functions, very often the Gompertz equation. This allows the estimation of an age‐independent mortality parameter representing baseline mortality (a combination of environmentally determined background mortality and initial individual vulnerability) and an age‐dependent parameter (usually interpreted to reflect physiological deterioration: Gaillard et al. 2017). Accurate interpretation of Gompertz parameters is difficult because both parameters include physiological and environmental components that inevitably interact and hence cannot be easily distinguished from each other (Abrams 1993; Ricklefs 1998; Caswell 2007; Burger 2017). Also, the measurement of senescence from the decline in phenotypic traits is susceptible to the effect of selective disappearance processes caused by heterogeneity in individual phenotypic quality. These processes mean that individuals that attain an old age are a nonrandom sample of the population and may include overrepresentation of “high quality,” physiologically more robust individuals that are able to successfully avoid natural hazards (Vaupel et al. 1979; van de Pol and Verhulst 2006; Hayward et al. 2013; Hämäläinen et al. 2014). Actuarial senescence is expected to be correlated with phenotypic senescence based on the assumption that physiological declines associated with ageing increase individual frailty (how likely negative environmental factors are to cause mortality in the individual). However, this relationship could be altered by the influence of selective disappearance and by environmental factors which affect physiological trait expression and survival differently. Empirical tests of the relationship between actuarial and phenotypic ageing trajectories will elucidate the extent to which cross‐sectional demographic and longitudinal phenotypic measures of senescence provide information on a common underlying process of biological ageing.

Existing studies usually rely on the analysis of capture–mark–recapture data to estimate actuarial (among‐individual) senescence (McDonald et al. 2014) and on analyses of age‐related changes in physical performance as a measure of phenotypic (within‐individual) senescence (e.g., Bouwhuis et al. 2009; Hammers et al. 2015). However, a comparative analysis of actuarial and phenotypic senescence is more powerful when a sample of multiple independent estimates of both is available. This is difficult to achieve with the long‐lived vertebrates that have been abundantly studied in the wild (Nussey et al. 2013; Bouwhuis and Vedder 2017). The statistical power of such an analysis is reduced when demographic senescence estimates are based on cohorts within overlapping generations because partially shared environmental histories mean that actuarial senescence estimates are nonindependent. This has been addressed using individual measures of mortality in the following year (Froy et al. 2018), but comparisons of actuarial and phenotypic senescence estimates from entire adult lifespans have not been attempted before (to our knowledge). We are aware of only two studies that compared the relationship between ageing trajectories of different traits and their relationship to lifespan in the field (Hayward et al. 2015) and in the lab (Briga 2016), reporting heterogeneous associations among ageing trajectories and lifespan, suggesting asynchrony of senescence across different traits. We build on long‐term vertebrate studies by estimating senescence in a wild insect population. As well as being much shorter lived than most species studied in the wild, the annual life‐history of most temperate insects means that each generation provides an independent sample (in the sense that individuals from discrete generations do not experience shared environmental conditions). This allows us to estimate demographic and phenotypic senescence across generations to examine the extent to which these measures are correlated.

Over 10 years (10 generations), we have been monitoring the survival and behavior of a natural population of the field cricket *Gryllus campestris*, living in a meadow in north Spain (Rodríguez‐Muñoz et al. 2010; Rodríguez‐Muñoz et al. 2011; Fisher et al. 2016; Fisher et al. 2018). Adult *G. campestris* are closely associated with burrows, which facilitates the recording of survival and behavioral data over individuals’ entire adult lives. By tagging every individual in the population and monitoring them 24 h a day using a network of digital video cameras (see “Methods” section), we have collected very precise demographic data as well as near‐continuous measurements of phenotypic trait expression over the course of each individual's life. This allows us to test the prediction that senescence will be apparent in both actuarial and phenotypic parameters over the adult lifespan of a few weeks. We then test the prediction that actuarial and phenotypic senescence will be positively correlated across generations.

## Methods

We monitored a wild field cricket (*G. campestris*) population in a meadow in northern Spain for 10 consecutive years. The *WildCrickets* meadow is managed in a similar way every year, with the grass being mowed in mid‐March and again in July–August. Between August and March, the grass is kept short with additional mowing. Weekly searches for burrows are made from February until the end of the breeding season sometime in July, when the last adult cricket dies. Each burrow is flagged with a unique number that will identify it for the whole breeding season. By mid‐to‐late April, usually before the adults start to emerge, we install between 64 and 133 infrared day/night cameras (the number of cameras increased from the initial 64 we had in 2006) that record the activity around each burrow entrance continuously. The cameras are connected to several computers provided with motion activated digital video recording software (Diginet, dvr-usa.com, replaced in 2011 with i‐Catcher, i-codesystems.co.uk) so that video is only recorded when movement is detected around the burrow.

A few days after emerging as an adult, we trap each individual using a device specifically designed for these crickets (see crickettrapping.wordpress.com). Each one is weighed (± 0.01 g), photographed and marked with a PVC tag glued onto the pronotum (ID), before being released back into the same burrow. The tag has a unique one to two character codes, which allows each individual to be identified on the video. For every individual, we also collect a sample of cuticular hydrocarbons (by gently rubbing the pronotum with filter paper around 100 times), an approximately 10 μL drop of hemolymph (sampled by piercing the membrane at the hind leg joint) and a small piece of the tip of one of the hind legs. These samples are later used to provide individual pheromone and DNA profiles.

Because the number of occupied burrows is often greater than the number of cameras, and adult crickets regularly move around the meadow occupying different burrows, we carry out direct observations to cover nonvideoed burrows. We do this by directly observing the occupants of every burrow that lacks a camera every one to two days. We record the ID of any adult present or whether a nymph is in residence. This allows us to accurately record adult emergence dates even in burrows that are not directly monitored at that particular time, as nymphs and recently emerged adults rarely move among burrows, and so the presence of an adult where there was a nymph the day before indicates an emergence. After the end of the season, we watch the videos and record all significant events (adult emergence, encounters between individuals, singing activity, matings, fights and their outcome, oviposition, predator attacks, movement of individuals around the meadow). The video data, together with the direct observations of burrows, are recorded in a database which currently includes >100,000 records. A weather station installed in the center of the meadow logs weather variables at 10 min intervals including measurements from seven additional temperature sensors located on the surface of the meadow (three sensors) and in simulated burrows (four sensors inside open‐end 15 cm long PVC pipes totally buried in the ground) at locations scattered around the meadow.

### ASSESSING VARIATION IN SENESCENCE IN WILD CRICKETS

Senescence can be detected (1) indirectly from the observation of an increase in the probability of mortality with age, presumed to result from physiological decline (known as actuarial senescence), or (2) directly through the effects of that decline on individual performance (phenotypic senescence). We used our observations of survival and individual traits to examine both processes across 10 generations.

#### Actuarial senescence

We quantified the rate of actuarial senescence using the R (ver. 3.4.0) package “BaSTA” which uses capture–mark–recapture data to fit and compare different age‐specific parametric survival models following a Bayesian approach (Colchero et al. 2012). A convenient aspect of studying senescence in insects is that adulthood can be precisely defined as the point at which an individual undergoes its final molt. The maximum adult cricket lifespan in our study population known to date is 84 days (average lifespan = 28.9 ± 17.9, mean ± SD, N = 1,135). We have unusually comprehensive capture–recapture data through our continuous monitoring program, allowing us to populate the capture–recapture matrix for BaSTA using the video and direct observations that provide daily individual recaptures (probability of recapture (phi) averaged across years = 0.51 ± 0.07, mean ± SD). Fitting each year separately (2006–2016, excluding 2014 in which video data extraction is incomplete), we found the two‐parameter Gompertz mortality distribution provided a fit with an *R* ^2^ of >0.92 in every year (mean *R* ^2^ across the 10 years was 0.95), and it was also the most widely supported model when comparing among exponential, Gompertz, Weibull, and logistic models (Table S1). The Gompertz model has two parameters: *b*_0_, the baseline mortality (the mortality rate independent of age), and *b*_1_, the age‐dependent mortality rate); *b*_0_ is the intercept and *b*_1_ is the slope of the natural logarithm of the mortality rate with age and is used as a measure of actuarial senescence (Gompertz 1825; Olshansky and Carnes 1997; Boonekamp et al. 2014). Because the purpose of our analyses is to compare the rates of actuarial senescence with phenotypic senescence, we selected the two‐parameter Gompertz distribution as the preferred model over more complex mortality distributions, whose parameters are more difficult to interpret in terms of actuarial senescence. Equally important, our comparison requires us to fit the same mortality distribution across years and the Gompertz distribution was the most widely supported model; in some years more complex mortality distributions were supported (Table S1), however their fit was only marginally better. There is some degree of error in our estimates of actuarial senescence, but this error is unbiased with respect to the estimation of actuarial senescence and is conservative in that its tendency is to decrease the statistical power to detect a pattern.

We ran four BaSTA simulations on the annual datasets, with 500,000 iterations, a burn‐in parameter of 50,000 and a thinning rate of 2000, which kept serial autocorrelation under 0.1.

#### Phenotypic senescence

As an indicator of phenotypic senescence, we used the effort males devote to produce energy intensive calling song (Hoback and Wagner 1997) to attract females for mating. To quantify calling effort, we recorded whether each monitored male sang or not with point samples taken over the 10 first minutes of every hour. For those 10 minutes, we watched at 1 minute intervals whether the male was singing or not. If at least one of those 10 samples per hour was positive, then the cricket was recorded as singing that hour. If singing was not observed for any of the 10 samples, he was recorded as not singing. For each studied male, this measure provided up to 24 binary samples per day throughout its life.

We carried out mixed effects logistic regression analyses using the *lme4* package (Bates et al. 2015) in R, to analyze the relationship between male calling effort (whether the male was calling) and age. To investigate the pattern of age‐specific calling effort we first fitted several spline functions of age with increasing complexity, and found that a quadratic relationship best fitted our data. Next, we used threshold model fitting (Douhard et al. 2017) to estimate the age of peak (threshold) calling and its AICc (Akaike information criterion with correction for small sample size) and confidence intervals. Unlike the peak of trait expression across age identified in a simple quadratic model (which minimizes variance across the entire distribution), the threshold model approach is designed to specifically identify the peak in which trait expression, which increases in early adulthood, begins to decline with the onset of senescence. The threshold model decomposes the age variable into pre‐ and postpeak age components, over a range of different peak ages. Support for a specific peak is then tested by evaluating the AICc values of the models over the range of peaks tested (ages 0–70 days). Following Burnham et al. (2011), we considered models to be equally supported when their AICc difference was <7. We ran individual optimizations for the discrete annual generations, facilitating subsequent analysis of the covariation between the estimates of postpeak age on calling effort (i.e., senescence when negative) and actuarial senescence, across generations. All models included individual ID as a random intercept effect. Pre‐ and postpeak age components, ambient temperature, and life span were included as fixed effects; this meant we had to exclude data from 2006 as temperature data were not recorded in that year. In this specific model structure, lifespan captures the among‐individual heterogeneity in maximum age, enabling interpretation of pre‐ and postpeak age variables as reflecting the longitudinal change in calling effort within individuals (note that the sum of the pre‐ and postpeak age is equal to age and hence that our model is similar to the commonly used longitudinal model approach based on age and lifespan). Random slopes of age for individual ID were not included because the computational demands of such a model structure with the large number of records in our dataset (*n* = 89,129) make this impractical. All age variables, including lifespan, were standard normal transformed (subtracting the mean across all observations from each value and dividing by the SD). This was required to reach correct model convergence.

## Results

### ACTUARIAL SENESCENCE

**Figure 1:**
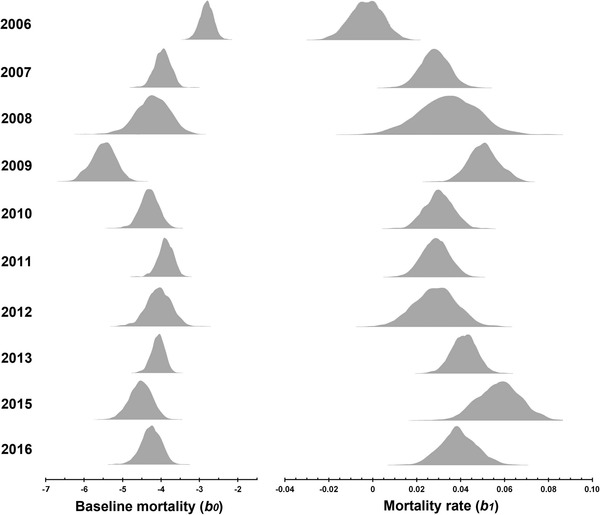
Posterior density distributions of baseline mortality (*b*_0_, left column) and actuarial senescence (*b*_1_, right column) in *Gryllus campestris* males, for a Gompertz model with simple shape fitted using the BaSTA R package (Colchero et al. 2012). Each row corresponds to a single year. Posterior means and 95 confidence intervals are available in Table 1.

The 95% credible intervals of our estimates of actuarial senescence (*b*_1_) did not include zero for any of the years, with the exception of 2006 (Fig. 1, Table 1). Our analyses therefore support the hypothesis that actuarial senescence is present in a short‐lived wild insect. There was substantial heterogeneity in mortality trajectories among years (Table 1). Indeed, the 95% credible intervals of the posterior distributions of baseline mortality (*b*_0_) and actuarial senescence (*b*_1_) completely fail to overlap in some of the possible pairwise comparisons among years, with the general pattern providing convincing evidence for differences among years in both baseline and age‐dependent mortality.

**Table 1:** Estimates and 95% credible intervals of baseline mortality (*b*_0_, the mortality independent of age) and age‐dependent mortality rate (*b*_1_, the coefficient for the effect of age on mortality), in a wild population of *Gryllus campestris* for 10 discrete generations

Year	*b*_0_—Baseline Mortality	*b*_1_—Age‐Dependent Mortality Rate	*pi*	*R* ^2^
2006	−2.817 (−3.126, −2.494)	−0.003 (−0.017, 0.010)	0.49	0.96
2007	−3.930 (−4.341, −3.582)	0.028 (0.016, 0.040)	0.48	0.92
2008	−4.210 (−5.033, −3.407)	0.034 (0.007, 0.059)	0.43	0.98
2009	−5.500 (−6.084, −4.940)	0.050 (0.037, 0.063)	0.58	0.92
2010	−4.317 (−4.760, −3.881)	0.030 (0.018, 0.041)	0.43	0.95
2011	−3.883 (−4.269, −3.534)	0.028 (0.017, 0.040)	0.47	0.95
2012	−4.046 (−4.668, −3.479)	0.028 (0.011, 0.045)	0.58	0.97
2013	−4.077 (−4.410, −3.742)	0.042 (0.030, 0.053)	0.54	0.93
2015	−4.532 (−5.113, −3.968)	0.057 (0.037, 0.076)	0.49	0.97
2016	−4.254 (−4.786, −3.748)	0.039 (0.023, 0.055)	0.65	0.96

Estimates of *b*_0_ and *b*_1_were calculated using BaSTA (Colchero et al. 2012) fitting a Gompertz model with simple shape, taking into account the recapture probability (pi). We also include a non‐Bayesian (i.e. least squares) goodness‐of‐fit estimate of the Gompertz model (*R* ^2^).

### PHENOTYPIC SENESCENCE

Threshold models provided clearly defined ages of peak expression, occurring around 15 days post adult‐emergence across years (Table S2, Fig. S1). Among years, the peak of calling varied from ages 12 to 19 days. We estimated the confidence intervals of the year‐specific peaks by taking the within‐year age range of thresholds that yielded a model fit with an AICc value <7 above the best fitting peak of that year (Burnham et al. 2011). This conservative approach nevertheless reveals an unexpected dichotomy between five years in which the peak is very close to 13 days and four years in which it is very close to 19 days. Apart from 2008, when the population was very small, these peaks have very tight confidence intervals (Table S2; Fig. S1). We examined potential relationships between the timing of the peak and trait expression trajectories before and afterward by comparing the estimates of pre‐ and postpeak age on calling activity between “early” and “late” years using a linear model with “early” versus “late” included as a factor. This test reveals that although there was a clear difference in the prepeak age trajectories between “early” and “late” years, with “late” years showing a reduced rate of increase (slope “early” − “late” = −0.339, *t* = −4.30, *P* = 0.004), we could not detect significant differences in the postpeak age trajectories in calling effort between the two categories of peak ages (slope “early” − “late” = −0.017, *t* = −1.43, *P* = 0.20).

**Figure 2:**
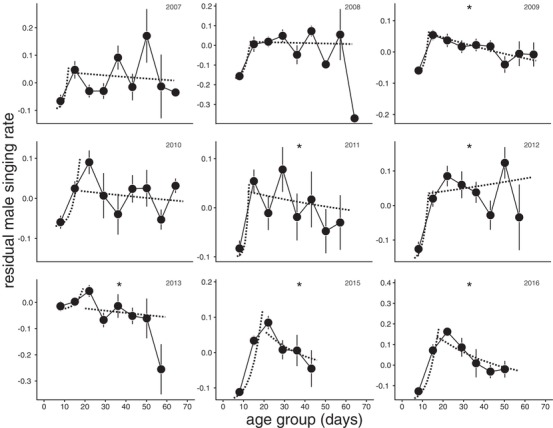
Age trajectories of male calling activity across the nine years of our study for which we had these data. Data points and error bars reflect the mean calling activity of age bins and their respective standard errors (note that the statistical analyses were done with the raw data, i.e., without binning of age). Dashed lines reflect the logistic regression lines of the pre‐ and postpeak age components as estimated by the best fitting threshold models.

Calling effort significantly increased with prepeak age in all years (Table 2, Fig. 2). Furthermore, we observed that there was a significant postpeak decline in calling effort with age in five of nine years, and a significant increase in calling effort with postpeak age in the year 2012 (Table 2, Fig. 2). Hence, we observed high heterogeneity in ageing trajectories of calling effort in which both peak age and subsequent postpeak ageing pattern substantially varied among the nine generations of our study.

**Table 2:** Relationship between age and the probability of calling in wild *Gryllus campestris* males calculated from a threshold model

			Fixed Factors					Random Factors	
Year	Samp		Int	Temp	Lifespan	Age Prepeak	Age Postpeak		ID
2007	9,971	Est	−14.09	0.30	0.001	0.69	−0.006	Var	0.54
		SD	0.63	0.009	0.008	0.051	0.003	SD	0.74
		*P*	***<0.001***	***<0.001***	0.865	***<0.001***	0.054	N	49
2008	3,098	Est	−13.93	0.41	−0.004	0.47	−0.002	Var	1.22
		SD	1.06	0.020	0.022	0.044	0.005	SD	1.10
		*P*	***<0.001***	***<0.001***	0.845	***<0.001***	0.643	N	13
2009	18,956	Est	−13.20	0.29	0.002	0.66	−0.012	Var	0.29
		SD	0.47	0.006	0.005	0.036	0.002	SD	0.54
		*P*	***<0.001***	***<0.001***	0.669	***<0.001***	***<0.001***	N	60
2010	7,036	Est	−11.27	0.25	0.000	0.37	−0.007	Var	0.53
		SD	0.51	0.009	0.008	0.024	0.004	SD	0.73
		*P*	***<0.001***	***<0.001***	0.965	<0.001	0.085	N	48
2011	5,570	Est	−19.04	0.44	0.019	0.87	−0.011	Var	0.75
		SD	1.09	0.018	0.012	0.084	0.005	SD	0.87
		*P*	***<0.001***	***<0.001***	0.133	***<0.001***	***0.028***	N	38
2012	7,414	Est	−12.07	0.25	0.014	0.50	0.007	Var	0.59
		SD	0.57	0.009	0.011	0.030	0.003	SD	0.77
		*P*	***<0.001***	***<0.001***	0.213	***<0.001***	***0.034***	N	26
2013	16,535	Est	−11.22	0.27	0.000	0.31	−0.012	Var	0.77
		SD	0.43	0.012	0.009	0.018	0.005	SD	0.88
		*P*	***<0.001***	***<0.001***	0.994	***<0.001***	***0.028***	N	77
2015	12,473	Est	−10.12	0.30	0.007	0.25	−0.055	Var	0.23
		SD	0.35	0.009	0.010	0.010	0.006	SD	0.48
		*P*	***<0.001***	***<0.001***	0.459	***<0.001***	***<0.001***	N	41
2016	8,076	Est	−8.93	0.21	0.006	0.32	−0.040	Var	0.40
		SD	0.36	0.009	0.009	0.013	0.004	SD	0.63
		*P*	***<0.001***	***<0.001***	0.496	***<0.001***	***<0.001***	N	31

We included ambient temperature (Temp) when each calling sample was recorded, Lifespan, Age Prepeak, and Age Postpeak as fixed factors, and individual identity (ID) as a random factor. The table shows the results per generation (Year). Samp, number of samples; Int, intercept; Est, coefficient estimates; SD, standard deviations; Var, variance; N, number of individuals. Coefficients with significant *P* values are highlighted in bold italics.

### COVARIATION BETWEEN ACTUARIAL AND PHENOTYPIC SENESCENCE

**Figure 3:**
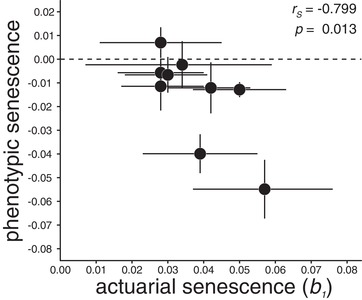
Relationship between the rate of actuarial senescence estimated from BaSTA and the slope of the effect of postpeak age on calling activity in *Gryllus campestris* males. Error bars denote the 95% confidence limits and *r_S_* denotes the Spearman rank correlation between the two metrics of senescence.

As described above, we found evidence for senescence in two commonly used ageing metrics—actuarial senescence and longitudinal ageing trajectories in our wild cricket population. We also observed substantial heterogeneity in both senescence metrics among the nine generations of our study. The relationship between actuarial senescence (*b*_1_) and postpeak ageing trajectories in calling effort can be seen in Figure 3. Years with higher actuarial senescence were also the years showing accelerated postpeak declines in calling effort (*r_S_* = –0.78, *P* = 0.013, Fig. 3). We also investigated covariation between actuarial senescence (*b*_1_) and the onset of senescence in calling behavior (i.e., the peak age), but the relationship between these two ageing metrics was not statistically significant (*r_S_* = 0.52, *P* = 0.15). Note that these results were robust with respect to the influence of baseline mortality *b*_0_, in the sense that when *b*_0_was included as covariate in a linear model (slope *b*_0_ = −0.02, *P* = 0.149), the partial correlation between *b*_1_and postpeak age remained statistically significant (slope *b*_1_ = −2.01, *P* = 0.014).

## Discussion

Our results support the handful of studies of lifespan in insects in natural or seminatural conditions that have found evidence for senescence over one (Bonduriansky and Brassil 2005; Kawasaki et al. 2008; Zajitschek et al. 2009a, 2009b; Carroll and Sherratt 2017) or two (Sherratt et al. 2010) breeding seasons. Senescence is a pervasive feature of our wild cricket population across multiple generations regardless of whether we measure it as demographic actuarial senescence or as a longitudinal decline in phenotypic performance within individuals. We observed actuarial senescence to be detectable in 9 of 10 years. However, there was also substantial heterogeneity in actuarial senescence among generations, similar to the differences between two seasons observed in damselflies (Sherratt et al. 2010). This heterogeneity among generations reveals that actuarial senescence estimates of single generations may provide limited information about senescence trajectories across generations, highlighting the importance of multigenerational studies. More importantly, the observed heterogeneity in actuarial senescence among generations is highly transient relative to the timescale of responses to natural selection. This reveals the strong impact of nonheritable factors, presumably dominated by environmental effects, on patterns of actuarial senescence.

The substantial heterogeneity observed in actuarial senescence trajectories across generations was also observed in our longitudinal analysis of phenotypic senescence, based on the effect of within‐individual age on the calling activity of males. Males showed strong age effects in terms of the rate at which they increased singing activity after becoming adult, and detectable age‐related declines in singing after they reached the peak in calling activity in five of the nine years that we could include in this analysis. As well as this variation in the rate of age‐related decline there was also variation in the onset of that decline. Examining the location of this peak age has the potential to provide insights into the process of senescence (Peron et al. 2010). It is intriguing that our nine years of phenotypic observations appear to fall into two groups with the peak age of calling activity occurring at either around 13 or 19 days (Table S2). We do not have a functional explanation for this dichotomy, and investigation of environmental effects on ageing trajectories, including the peak age, is a substantial endeavor in its own right. However, we were able to use our identification of peak ages to establish that the observed variation in the postpeak age trajectories are not predominantly side effects of the differences in the timing of the onset of senescence because no relationship between peak age and the rate of postpeak age trajectories was apparent.

The mechanisms underpinning environmental variation in demographic patterns of actuarial senescence remain elusive, despite being an important topic of ageing research. Individual differences in lifespan may be caused by a multitude of factors including among‐individual heterogeneity in phenotypic quality and within‐individual variation in the rate of biological ageing (Speakman 2005). It will be crucial to determine the extent to which actuarial senescence reflects either of these two lifespan components to interpret patterns of actuarial senescence in the context of ageing. We are aware of only a few studies that addressed this topic (de Magalhães 2006; Briga 2016) and to our best knowledge no such study exists in the wild where the impact of environmental conditions on phenotypic quality selection may even be more pronounced.

By directly comparing patterns of phenotypic and actuarial senescence among years we identify a positive correlation between these measures (Fig. 3). This indicates that, although actuarial senescence is the outcome of combined within‐ and among‐individual processes, the signal from within‐individual declines occurring with age remains dominant in patterns of variation among generations. Our finding suggests that the widespread practice of interpreting measures of actuarial senescence as indicative of phenotypic senescence is justified. However, recent studies suggest that there may be variation in ageing trajectories among different performance traits (Hayward et al. 2015; Briga 2016), implying that correlations between actuarial and phenotypic senescence patterns may depend on the traits selected for such comparison. It is also worth noting that an earlier analysis (not shown) in which we identified the location of the peak in calling effort by simply using the peak identified in a quadratic model, completely failed to identify a relationship between actuarial and phenotypic senescence. This indicates that methods for correctly identifying the region of the lifespan over which senescence occurs is an important aspect of quantifying age‐related declines in performance (Douhard et al. 2017).

The substantial heterogeneity we observed in rates and timing of both actuarial and phenotypic senescence among years highlights the importance of incorporating environmental factors into theories of senescence (Furness and Reznick 2017). The precise climatic and biotic factors that impinge upon ageing will inevitably be taxon‐specific. For temperate insects they are likely to include climatic variables (such as the ambient temperature during the preadult overwintering period, rainfall during the breeding season, levels of insolation, etc.), biotic variables (the impact of particular predators in our meadow varies considerably among years, the composition of plant species varies, etc.) and demographic parameters (population size, mean emergence date, etc.). Our study establishes the potential for individual‐level observations of both phenotypic and actuarial senescence across nonoverlapping generations in wild invertebrates. Systems such as this hold the potential for further insights into the relationships between actuarial senescence, phenotypic senescence, and environmental factors that impinge upon them.

Associate Editor: S. Sumner

Handling Editor: M. R. Servedio

## Supporting information


**Table S1**. Delta DIC values per year estimated from BaSTA when fitting various mortality models and shapes to the male survival data of a wild population of *G. campestris*.
**Table S2**. Annual peak age of calling activity in wild *G. campestris*.
**Figure S1**. Age thresholds of male calling activity (i.e. the age of the peak of calling activity using a quadratic age relationship) and their AICc values.

## Data Availability

Should the manuscript be accepted, the data supporting the results will be archived in an appropriate public repository such as Dryad or Figshare and the data DOI will be included at the end of the article.
