# Supplementary material for: Comparing individual and population measures of senescence across 10 years in a wild insect population
Source: Evolution. 2019 Jan 10;73(2):293–302. doi: 10.1111/evo.13674 (PMC6590638; doi:10.1111/evo.13674)
Supplement: Supplementary file 1 — Table S1. Delta DIC values per year estimated from BaSTA when fitting various mortality models and shapes to the male survival data of a wild population of G. campestris. Table S2. Annual peak age of calling activity in wild G. campestris. Figure S1. Age thresholds of male calling activity (i.e. the age of the peak of calling activity using a quadratic age relationship) and their AICc values. [file EVO-73-293-s001.pdf]

Supporting information

Table S1: Delta DIC values per year estimated from BaSTA when fitting various mortality models and shapes to the male survival data of a wild population of *G. campestris*. The model with the lowest DIC value each year is scored as 0, with the values of all the other years showing the difference in relation to that lowest value. Blank cells indicate tests for which convergence was not attained.

Model	Shape	2006	2007	2008	2009	2010	2011	2012	2013	2015	2016
Exponential	-	2.3	21.8	7.3	63.1	28.3	24.6	565.9	68.9	37.0	21.6
Gompertz	simple	6.4	0.0	0.0	1.5	0.0	2.9	0.0	17.6	5.0	3.3
	Makeham	3.2		11.1	0.0	2.4	0.0	16.6		7.3	18.6
	bathtub	10.0	5.9	5.1	3.7	13.0	8.2	1.0	32.9	11.1	12.7
Weibull	simple	3.4	3.0	0.2	18.9	3.5	3.6	3.0	1.3	1.3	3.8
	Makeham	4.0	2.2	3.1	2.9	6.3	0.7	1.8	14.0	0.0	7.5
	bathtub	11.0	4.7	3.4	6.9	7.5	5.7	3.1	6.6	5.8	6.5
Logistic	simple	6.1	4.4	0.4	5.5	8.5	10.8	1.1	0.0	10.6	0.0
	Makeham	0.0	3.4	2.8	0.4	10.2	1.6	5.6	8.8	5.2	12.2
	bathtub	11.5	8.0	9.1	6.4	9.5	9.6	4.3	11.4	8.9	11.8

Table S2: Annual peak age of calling activity in wild *G. campestris*. The age range reflects the confidence interval per year based on delta AICc <7 (i.e. models within this threshold age range were considered to support the data equally well). Different letters in the 'Significant differences' column denote years with non-overlapping confidence limits.

year	Threshold age	Significant differences
2007	12 (12-12)	a
2008	13 (11-26)	ab
2009	12 (12-12)	a
2010	18 (17-19)	b
2011	12 (12-13)	a
2012	13 (13-14)	a
2013	18 (17-20)	b
2015	19 (18-19)	b
2016	17 (17-18)	b

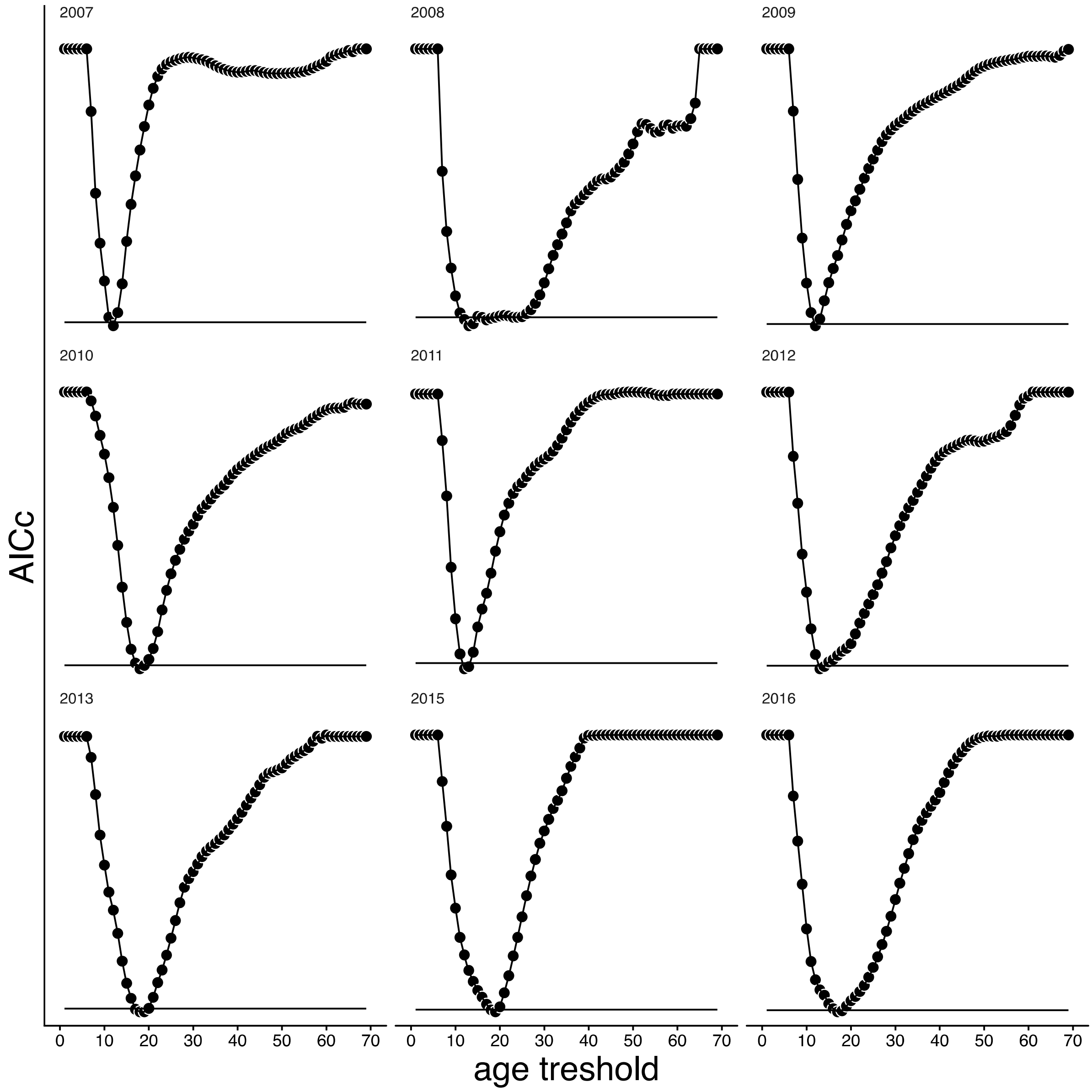

Figure S1. Age thresholds of male calling activity (i.e. the age of the peak of calling activity using a quadratic age relationship) and their AICc values. Horizontal lines denote AICc differences of 7 above the best fitting age threshold and hence models with ages resulting in AICc values below this line are considered to be equally well supported by the data.
